# Global bibliometric analysis of conceptual metaphor research over the recent two decades

**DOI:** 10.3389/fpsyg.2023.1042121

**Published:** 2023-02-10

**Authors:** Xia Zhao, Yi Zheng, Xincheng Zhao

**Affiliations:** ^1^School of Foreign Languages, Jiangsu University of Science and Technology, Zhenjiang, China; ^2^Center for Applied English Studies, The University of Hong Kong, Hong Kong, Hong Kong SAR, China

**Keywords:** bibliometric analysis, interdisciplinary study, quantitative and qualitative, evaluation, research trends

## Abstract

Conceptual Metaphor has been a prevalent theme in the linguistic field for the recent twenty years. Numerous scholars worldwide have shown interest in it and published many academic papers from various stances on this topic. However, so far, there have been few rigorous scientific mapping investigations. With the help of bibliometric analysis tool, we selected 1,257 articles on Conceptual Metaphors published from 2002 to 2022, as collected in the Web of Sciences Core Collection database, from unique cognitive perspectives. The global annual scientific output of Conceptual Metaphor, including the cited articles, sources, keywords, and research trends, will be examined in this study. The most notable findings of this study are the following. First, there has been an upward trend in Conceptual Metaphor research over the last two decades. Second, the five most prominent research groups on Conceptual Metaphors are in Spain, the United States of America, China, Great Britain, and Russia. Third, future research on Conceptual Metaphors may focus on corpus linguistics, neurolinguistics, psychology, and critical discourse analysis. The interdisciplinary study may enhance the growth of Conceptual Metaphors.

## Introduction

There is an enormous amount of literature in numerous research areas in the times of big data (e.g., Faust et al., 2018; Alnajem et al., 2021; Bashir et al., 2021; Abdul et al., 2022). Much research, however, is dispersed and difficult to compile in an orderly and visible manner. Therefore, finding specific literature quickly and accurately relevant to the research issue has always been challenging. For example, since Lakoff and Johnson proposed conceptual metaphors (CM) (Lakoff and Johnson, 1980), academic papers on the growth of CM have undoubtedly increased over the past decades, and they have helped to advance numerous facets of CM study. However, keeping up with everything published instantaneously becomes more problematic. In terms of CM research, there are increasing studies on linked subjects, and various reviews have been conducted (e.g., Allahmoradi, 2018; Holyoak and Stamenkovic, 2018; Bundgaard, 2019; Gandolfo, 2019; Tohidian and Rahimian, 2019; Kövecses, 2020; Bearman et al., 2021; Jensen et al., 2021; Abdul et al., 2022). However, these studies mainly focused on qualitative analysis. So far, little research has been done on a general picture of CM research. Therefore, this study uses Bibliometrix metrology software, a statistical package called R (Biblioshiny), to visually analyze academic articles on CM over the recent two decades. The prevalent topics in different areas on the present research status, research themes, and future research directions in CM will provide references for scholars to research CM and predict its future direction.

This study is a valuable resource for academics and researchers working in the CM field. Beginners interested in CM can be offered the information required to start their research. Experienced CM researchers can familiarize themselves with the advances in the field and promote collaboration and networking between institutions and authors.

## CM research (2002–2022): A bibliometric analysis

### Bibliometric tool

Bibliometrics is an open-source tool for quantitative research in scientometrics^1^. It is a unique tool using the R programming language for statistical computation and graphics following a logical bibliometric process. The bibliometric tool is controlled by Bibliometrix and its web-based graphical interface based on Web of Science (WoS), Scopus, and Dimensions data (Aria and Cuccurullo, 2017). The interface is spontaneous and well-systematized, and the main menu is separated consistently with the Science Mapping Analysis system. The set menu performs analysis from eight categories: data sets, sources, authors, documents, clustering, intellectual structures, conceptual structures, and social structures in Biblioshiny. Several document formats can be transmitted, maps can be transferred to Html or Pajek, and tables can be saved as pdf, excel, or printed. The R bibliometrics package was used to analyze data, which gives more objective and dependable assessments than other methods. By providing a structured analysis of a large body of knowledge, bibliometrics becomes helpful when there is an excellent volume of new information, conceptual developments, and data to process.

Today, the bibliometric tool is increasingly utilized in numerous fields. An amount of research has been conducted (Ellegaard and Wallin, 2015; Donthu et al., 2021; Efron et al., 2021), including tsunami research (Chiu and Ho, 2007; Jain et al., 2021), the circular economy (Geissdoerfer et al., 2017; Bui et al., 2020; Luis and Celma, 2020; Alnajem et al., 2021), green supply chain management (Fahimnia et al., 2015; Amirbagheri et al., 2019), deep learning for healthcare applications (Faust et al., 2018; Saheb et al., 2021; Zhu et al., 2021), environmental hypothesis (Sarkodie and Strezov, 2019; Bashir et al., 2021; Hashemi et al., 2022), and COVID-19 research (Verma and Gustafsson, 2020; Yan et al., 2021). Waltman et al. (2010) assert that scholars frequently mix mapping and clustering techniques when analyzing bibliometric networks. They employ bibliographic data from publishing databases to build structural pictures of scientific domains (Zupic and Čater, 2015). The growing number of papers using bibliometric analysis across all disciplines suggests that it meets the desire of researchers who want proper research based on a wealth of literature.

Attributable to its reliable and scalable statistics, the bibliometric tool has compelling features and is becoming increasingly important in research. In contrast to other methods, it can introduce a systematic, transparent, and repetitious review procedure based on statistical assessments of science, or scientific activity. It adapts when the focus on empirical inputs results in extensive, dispersed, and contentious research streams, making it a particularly effective tool for science mapping (Aria and Cuccurullo, 2017). This tool allows us to infer CM research trends and various themes researched, identify shifts in the boundaries of disciplines, find the most prolific scholars and institutions, and provide the large picture of prevailing research. Popular and thorough bibliometric analysis allow us to sift through and make sense of massive amounts of scientific data (Donthu et al., 2021, p. 285). This study aided us in analyzing the nuances of the CM research field’s evolutionary process and sheds light on its developing regions.

### Conceptual metaphor research

In cognitive linguistics, conceptual metaphor (CM) refers to comprehending one thought or abstract concept in terms of another. Since 1980, theoretical clarification of CM has been the topic of extensive studies and lengthy introductions by many researchers (e.g., Lakoff, 1987; Lakoff and Turner, 1989). They showed great interest in CM research. According to Lakoff and Johnson, the mechanism of CM is as the following. “In a metaphor, there are two domains: the target domain and the source domain; in addition, metaphoric mapping is multiple. Two or more elements are mapped to two or more other elements. Image-schema structure is preserved in the mapping.” (Lakoff and Johnson, 2003, p. 266). They argue that metaphor is internally structured, and its meaning is derived from transferring specific characteristics from the original to the new field. As the surface manifestation of this mapping, the metaphorical expressions might be words, phrases, or whole sentences. The source domain is a somewhat tangible or, at the very least, strongly organized realm that often derives from our everyday experience. However, the target domain is where the metaphor is applicable and originates, a somewhat more abstract or unorganized field using unknown notions. Metaphor is the process of understanding one idea in a target domain *via* the other in a source domain. For instance, in the frequent metaphorical expression LOVE IS A JOURNEY (Lakoff and Johnson, 2003, p. 45), the target domain “LOVE” is abstract and difficult to construe. “JOURNEY” is the realm of the source. “LOVE” somewhat maps the “JOURNEY” structure in the following procedure: departure, on the way or lost way, and destination. As the conceptual metaphor is a mental construct, it is only meaningful when represented in more tangible elements. Therefore, this sentence consists of many metaphorical analogies that form a unified inner structure. “LOVE” and “JOURNEY” are strongly connected in this sense. The idea of “love as a journey” shapes the conceptualization of love itself. Even though “love” might be understood in ways other than a journey, we use this comparison to impact our understanding and attitude toward LOVE. This approach is how we may see, experience, participate in, and refer to LOVE IS A JOURNEY. Metaphor infuses our language, daily lives, and actions. Because the mind is experienced, our cognition is experiential. Remarkably, human cognition derives from our personal experience of the external world, shaping our perspective on the outer world.

Undeniably, the growth of academic papers on CM has contributed to the advancement of CM research. Numerous scholars have conducted a significant amount of research into CM from various perspectives, such as psycholinguistic metaphor research (Murphy, 1996; Gibbs, 2013; Qiu et al., 2022); deliberate metaphors and embodied simulation research (Gibbs, 2006; Cuccio and Steen, 2019; Cuccio et al., 2022); conceptual conflicts in metaphors and translation (Prandi, 2017; Rizzato, 2021, 2022); corpus-based metaphor research (Sinclair, 1991; Charteris-Black, 2000, 2004; Semino, 2002; Deignan and Potter, 2004; Allen, 2006; Fabiszak, 2007; Tissari, 2010; Shutova et al., 2013; Burgers and Ahrens, 2018; Zhao and Zhou, 2019; Zhao et al., 2019, 2020; Silvestre-López, 2020; Bosman and Taljard, 2021; Kazemian and Hatamzadeh, 2022), critical metaphors in discourse analysis (Charteris-Black, 2004; Ferrari, 2007; Musolff, 2012) and metaphors in classroom teaching (Thomas and McRobbie, 2001; Andreou and Galantomos, 2008). There is also increasing research on various reviews of CM research (e.g., Allahmoradi, 2018; Holyoak and Stamenkovic, 2018; Bundgaard, 2019; Gandolfo, 2019; Tohidian and Rahimian, 2019; Kövecses, 2020; Bearman et al., 2021; Jensen et al., 2021; Abdul et al., 2022).

However, researchers need help to pinpoint the research status and anticipate research trends rapidly and correctly. Keeping up with articles published instantly also becomes increasingly challenging. By using a bibliometric analysis, this knowledge map will be an invaluable resource for beginning researchers to learn more about information and study results to start their investigation as soon as possible. Additionally, this study will identify future research gaps and find potential cooperators for seasoned scholars. In addition, this study will provide some rating agencies with a trustworthy benchmark to assess the effectiveness of authors, institutions’ sources, and nations in CM research. Nevertheless, there has not been a thorough visual of CM studies so far. The bibliometric analysis of Bib text provides extra data statistics, including author, affiliation, and keyword (Fahimnia et al., 2015). Therefore, this study will fill the gap by analyzing the state of CM’s research over the past 20 years, its current focus areas, and future research directions.

## Methodology

### Research questions

With the bibliometric tool, this study aims to provide an overall picture of CM research over the recent two decades and address the following three questions:

(1) What was the basic information about the development of international CM research in the past two decades?
(2) What is the present situation, including yearly scientific advancements, subject orientations, most renowned authors, and the most pressing issues in CM research?
(3) What predictions may be made regarding its future development based on a bibliometric study?

### Data source

All the data in this study were obtained from WoS Core Collection. It is the platform’s flagship resource, covering over 21,000 peer-reviewed, high-grade scientific articles (containing Open Access journals), more than 205,000 conference proceedings, and more than 104,000 editorially selected book^2^.

It offers more reliable journal coverage of scholarly published articles (Birkle et al., 2020) than any other databases like Scopus and Google.

### Data collection

This study discerningly chose the WoS that confined the data from 2002 to 2022. The literature data gained comprised the whole archives, such as the author’s name, source year, abstract, keywords, citation frequency, DOI number, and references in the article. Data collection consisted of three stages. The first was data reclamation. We prudently chose the papers and early access collected in the arts and humanities citation index (AHCI) and the SSCI to evaluate research questioning. We scrutinized the principal articles consistent with the research topic. The second step was data scrubbing. We sifted papers discreetly to avert data duplication. In the third step, documents were downloaded and compacted. We downloaded 1,000 files the first time and 257 the second time. Subsequently, the two files were compacted using bibliometric instruments. Currently, diverse instruments are accessible to present visual studies, such as CitNetExplorer, CiteSpace, and VOSviewer. This study selected a Biblioshiny program to obtain an overall visual picture of CM study in the past two decades because it has unique features. The set menu in Biblioshiny presents analysis from source, author, and document dimensions. Additionally, this menu offers conceptual, intellectual, and social knowledge structures. Maps can be exported to HTML or Pajek, tables can be copied to the clipboard or saved as Excel or PDF files, and maps can be printed. We analyzed the data using the Rstudio software and the bibliometric R-package version 4.2.0. The bibliometric analysis was first enabled in the R environment using the following command code:

install. Packages (“bibliometrix,” dependencies = TRUE)
library (bibliometrix)
biblioshiny()

The Biblioshiny web interface was presented once the Google Chrome browser started with the above code. Raw WoS data were imported and analyzed using the Biblioshiny. We then went on to describe and evaluate the critical results of the study, which were shown with the statistics and pictures. This study employed pertinent authors, institutions, countries, articles, top highly cited publications, keyword co-occurrence, word clouds, thematic maps, trend topics, and conceptual framework to answer the above three research questions of the study.

## Results and discussion

### Position of CM research in the past two decades

Table 1 presents the key findings of the entire CM research from January 1, 2002, to July 10, 2022. In total, 1,257 documents were present. There were 317 sources for the CM research, including books, journals, and other materials. The average number, like years from publication, citations per document, and citations per year per document, were 7.49, 8.228, and 0.7505. The number of references cited in the studies reached 33,265, demonstrating the popularity of CM research over the previous two decades. The 956 papers represented the most significant categories of published documents. The author’s keywords and the plus were 3,130 and 846, respectively, in terms of the document contents. It demonstrates the variety of topics covered by CM research and 1,544 contributors to CM studies from 2002 to 2022. There were 613 authors of single-authored documents and 931 authors of multi-authored documents. The single-authored documents are 776, showing that the scholars are highly interested in this area. The documents per author were 0.814, while authors per document, co-authors per document, and collaboration index were 1.23, 1.59, and 1.94. It indicates that more scholars concentrate on CM research, and the direction conducted by multiple authors was the most important means for CM research in the past two decades (see Table 1).

**TABLE 1:** Main information about data.

Description	Results
**Main information about data**	
Timespan	2002: 2022
Sources (journals, books, etc.)	317
Documents	1,257
Average years from publication	7.49
Average citations per documents	8.228
Average citations per year per doc	0.7505
References	33,265
**Document types**	
Article	956
Article; early access	19
Article; proceedings paper	21
Book review	40
Editorial material	13
Proceedings paper	198
Review	9
Review; early access	1
**Document contents**	
Keywords plus (ID)	846
Author’s keywords (DE)	3,130
**Authors**	
Authors	1,544
Author appearances	1,993
Authors of single-authored documents	613
Authors of multi-authored documents	931
**Authors collaboration**	
Single-authored documents	776
Documents per author	0.814
Authors per document	1.23
Co-authors per documents	1.59
Collaboration index	1.94

### Annual scientific production

**FIGURE 1:**
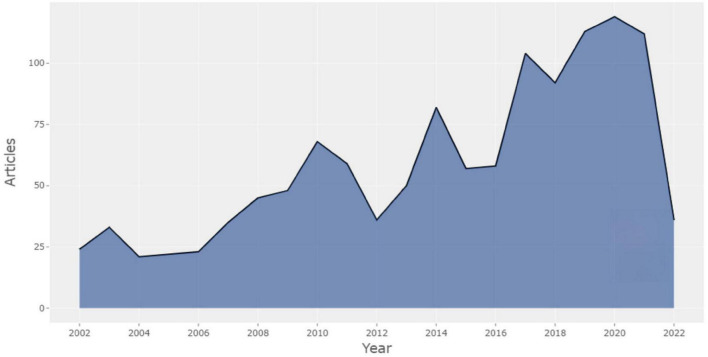
Annual scientific production.

A highly intriguing phenomenon has been discovered in annual scientific production. Figure 1 depicts the dynamics of document creation. The number of papers published annually was balanced from 2004 to 2006 and steadily rose from 2002 to 2022. The most productive year for the output was 2020, with 119 publications, including *Gender, Ideology, and Conceptual Metaphors: Women and the Source Domain of the Hunt* (Maestre, 2020) and *Conceptual Metaphors Leading to Some Names of Anger in the Indo-European Languages* (With Focus on the Romance Languages) (Georgescu, 2020). Notably, this number has steadily increased, with a yearly growth rate of 2.05 percent. The number of studies on CM and the total number of articles published has expanded significantly over the past 5 years. The yearly variation in literature production may represent the shift in the research subject, research interest, depth, and future development direction. CM has been a prevalent topic in the linguistic field over the past two decades, and it may continue to be a future topic in this field. In other words, the CM in Cognitive Linguistics has garnered great academic interest over the past two decades.

### Analysis of cited documents

#### Average annual citations

**FIGURE 2:**
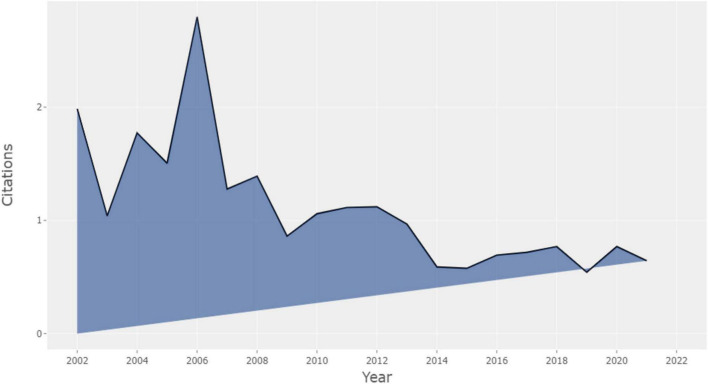
Average article citations per year.

In Figure 2, we can see the typical annual number of article citations. The most significant number of citations was 2,796 in 2006, while the least was 0.546 in 2019. Typically, the yearly average citation rate of recent articles is low. There is a surprising phenomenon: the citation rate of CM articles in 2006 reached a peak, but the publications in 2006 were low. Therefore, the citation rate may be more relevant to the articles’ quality and themes rather than their quantity. The top four average annual citations articles in 2006 are the following, *Metaphor Interpretation as Embodied Simulation* (Gibbs, 2006), *The Emergence of Metaphor in Discourse* (Cameron and Deignan, 2006), *Does Understanding Negation Entail Affirmation: An Examination of Negated Metaphors* (Hasson and Glucksberg, 2006), *Metaphoric competence, Second Language Learning, and Communicative Language Ability* (Littlemore and Low, 2006). The constant average citation per year after 2014 in Figure 2 shows that CM research has lately had a stable development.

#### Most global citated articles

**FIGURE 3:**
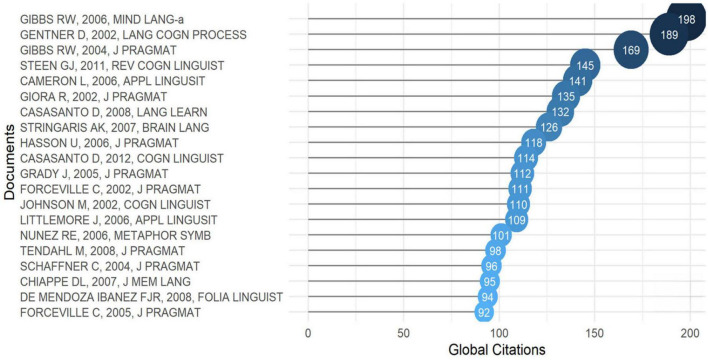
Most global cited documents.

Figure 3 shows CM’s top 20 most globally cited documents from 2002 to 2022. According to Figure 3, Gibbs’s article *Metaphor Interpretation as Embodied Simulation* (Gibbs, 2006) was most passionately cited with 198 citations, hierarchical first among all other documents. In this study, Gibbs (2006) claims that part of our ability to make sense of metaphorical language, both individual utterances and extended narratives, resides in the automatic construction of a simulation whereby we imagine performing the bodily actions referred to in the language. *As Time Goes by: Evidence for Two Systems in Processing Space → Time Metaphors* (Gentner et al., 2002) has 189 total citations and demonstrates that individuals employ spatial metaphors in temporal thinking. The metaphoric systems’ status implications are examined in it. With 169 citations, Gibbs et al. (2004) review the empirical evidence and discuss the methodological strategies employed by linguists and psychologists seeking connections between embodiment and CM. Subsequently, *The Emergence of Metaphor in Discourse* (Cameron and Deignan, 2006), *Literal vs. figurative language: Different or Equal*? (Giora, 2002). Other significant subjects of CM research are the relationship between CM and metonymy, studying psychology and politics of metaphors, and CM based on language theory. Most of the literature that generates the most citations has been published for more than ten years, indicating that the topic and authority of the publication may be the reason for the number of citations. Figure 3 also shows that the years of highly cited literature on CM were 2006, 2002, and 2004, representing that CM has made a breakthrough in development during these years. Besides, Figure 3 suggests that a longitudinal study of how CM works over time is crucial to scrutiny. In general, the more citations an article has, the more influential it will be in the CM field. Moreover, Figure 3 proves that Gibbs’ article published in 2006 was the most relevant document contributing to the CM research. The research of CM is closely related to human psychology and cognition, and it may be more concerning and exciting to scholars when they conduct empirical research.

#### Source growth

**FIGURE 4:**
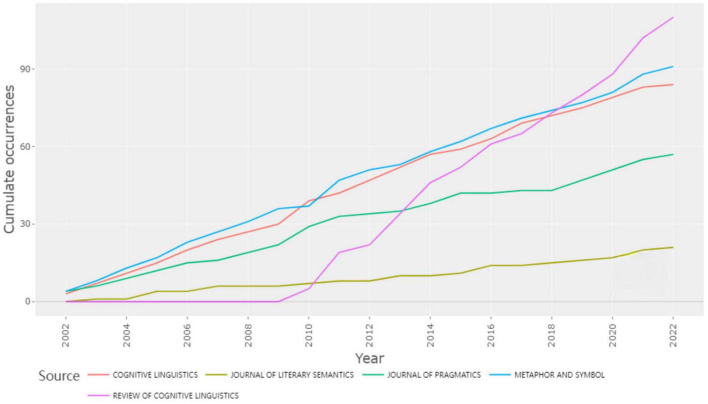
Source dynamics.

Figure 4 depicts the source dynamics of the top five journals from 2002 to 2022. Regarding the number of articles, Figure 4 shows a significant increase trend, with the peak in 2022 and the lowest in 2002. The corresponding maxima are the following: *Review of Cognitive Linguistics, Metaphor and Symbol, Cognitive Linguistics, Journal of Pragmatics*, and *Journal of Literary Semantics*. The increase in sources illustrates the main application areas of CM research over the past two decades and its multidisciplinary development trend. As indicated in Figure 4, *Journal of Review of Cognitive Linguistics* has published CM articles in recent years with the highest growth rate, particularly between 2012 and 2022. This Journal’s quick expansion indicates that several experts enthusiastically pursue the debate and research on CM. Despite being among the top five, as shown in Figure 4, *Journal of Literary Semantics* had a steadily increasing number of CM papers published from 2002 to 2022. Only 21 articles were published in this journal in 2022, but there were 110 articles in *Reviews of Cognitive Linguistics*. The number indicated that the Journal’s discussion subject might diverge from the study category of CM. From 2002 to 2022, we judged from the growth trend of article sources that CM’s research showed a sound momentum of rapid progress over the last two decades.

### Authors, affiliations and countries

#### Prolific authors

Gibbs was the most significant researcher, who published 17 articles and ranked first in document number on CM, concentrating on the embodied metaphor and mapping in cognitive linguistics in terms of the author’s output from 2004 to 2022. Gibbs’s articles *On the Psycholinguistics of Sarcasm* and *How to kick the Bucket and not Decompose: Analyzability and Idiom Processing*, with more than 200 references to *Spilling the Beans on Understanding and Memory for Idioms in Conversation*. Gibbs is committed to studying embodied metaphors and mapping in cognitive linguistics and makes significant contributions to the CM research. Following Gibbs, Yu published nine documents mainly scrutinizing the spatial subsystem of moral metaphors in English. De Mendoza Ibanez represents Spanish research on CM with nine articles. He explores metaphors concerning cognitive prominence and conceptual interaction issues.

Moreover, he deals with the problems of constraints on metaphor and proposes three complementary kinds of constraints. Over the past two decades, these three authors were the most productive and essential in the CM research field. They are vital scholars, and their views may provide a theoretical and practical framework for further research.

#### Most relevant affiliations and countries

Most relevant affiliations can present the top most relevant affiliations according to the number of articles about CM. The University of La Rioja, the University of California, Santa Cruz, the University of Birmingham, Castile La Mancha University, and Guangdong University of Foreign Studies were the five most relevant affiliations by producing 68, 30, 28, 28, and 25 articles in the past two decades, respectively. They are also the bases for linguistic research. The result derived from the cooperative efforts of various institutions and was focused on CM subjects.

**FIGURE 5:**
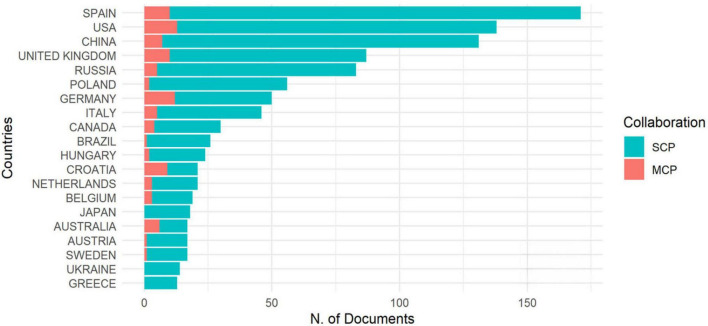
Corresponding author’s country. Multiple country publications (MCP), the number of papers co-authored with authors from other countries; SCP, the number of papers co-authored by authors of the same nationality. The MCP ratio represents the ratio of international cooperation.

Figure 5 shows the countries of the top 20 corresponding authors. The collaboration of authors of the same nationality was far more than that between nations. According to Figure 5, the top 20 countries contributed a lot to the research of CM during the past two decades. Among them, Spain contributed the most to it, with 171 publications, followed by the United States (138), China (131), the United Kingdom (87), and Russia (83). These countries occupied the top five in the WoS Core collection. The result showed that CM research attracted the attention of researchers in these countries in the past two decades. To some extent, the publications of these authors will benefit CM’s future development.

Table 2 lists countries, average article citations, and total citations for the top 20 relevant nations. Table 2 shows that the United States made the most considerable contribution to CM research, with 3,203 total citations and an average article citation rate of 23.210. The United Kingdom and Spain came next with 1,378 and 1,112 total citations, respectively. It implies that scholars from the top three nations show great interest in CM research. A particular topic of study was directly tied to the context of the nation. Therefore, the United States, the United Kingdom, and Spain contribute to CM research development and associated linguistic issues with more attention. The fact that more nations, including the Netherlands, China, and Italy, are paying attention to CM research shows how prevalent it has become over the past two decades.

**TABLE 2:** Most cited countries.

Country	Total citations	Average article citations
USA	3,203	23.21
United Kingdom	1,378	15.839
Spain	1,122	6.561
Netherlands	590	28.095
china	446	3.405
Italy	307	6.674
Hungary	275	11.458
Canada	266	8.867
Israel	257	28.556
Germany	241	4.82
Austria	227	13.353
Sweden	194	11.412
Denmark	171	24.429
Belgium	136	7.158
Poland	134	2.393
New Zealand	118	23.6
Russia	115	1.386
Japan	104	5.778
Greece	102	7.846
Australia	86	5.059

We can also see from Table 2 that although the corresponding authors of CM research in the United States ranked second, their citation rate ranked first. Similarly, the corresponding authors in the UK rank fourth, while the citation rate of their authors ranks second. Therefore, the number of correspondents does not have a one-to-one proportional correspondence with their citation rate. From this, we can infer that the citation rate may relate to the article’s quality and themes.

#### Conceptual structure

**FIGURE 6:**
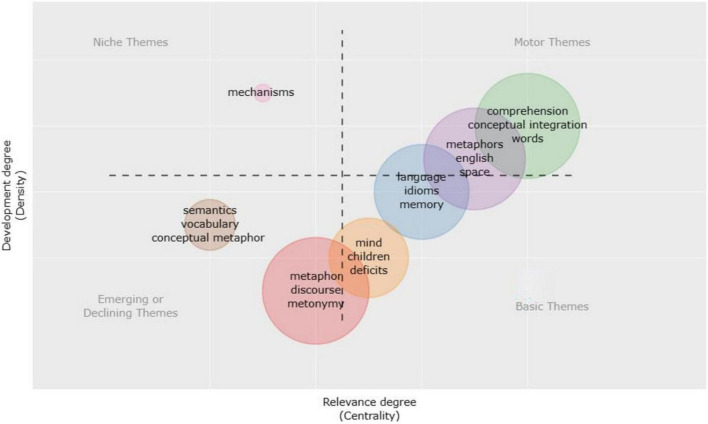
Thematic map.

Figure 6 presents the current status of thematic groups in CM research. Thematic maps illustrate a particular topic and help reveal geospatial patterns and relations (Schaab et al., 2022). A thematic map is separated into four quadrants grounded on the degree and density of centrality. High density and centrality in the upper right quadrant represent well-developed Motor Themes in the CM research area. In this quadrant, many themes comprised the emphasis and center of CM research, such as “comprehension,” “conceptual integration,” “words,” “metaphors,” “English,” “space,” and “language.” These themes had outstanding growth in the past two decades. The second quadrant’s high density and low centrality imply niche themes with good development prospects but a limited influence on the research field. Although scholars have created a “mechanisms” research group, its prospects are unsure. Subject clusters have poor centrality and density in the lower-left quadrant. It implies that different types of “semantics,” “vocabulary,” “metaphor,” “discourse,” “conceptual metaphor,” and “metonymy” are marginalized. It suggests they are new or waning themes. The fourth quadrant’s high centrality and low density indicate that “mind,” “children,” “deficits,” “idioms,” and “memory” are the primary topics in CM. Their theoretical systems are more thorough and mature, and these core topics may provide the theoretical foundation, reasoning, and technique for CM research.

**FIGURE 7:**
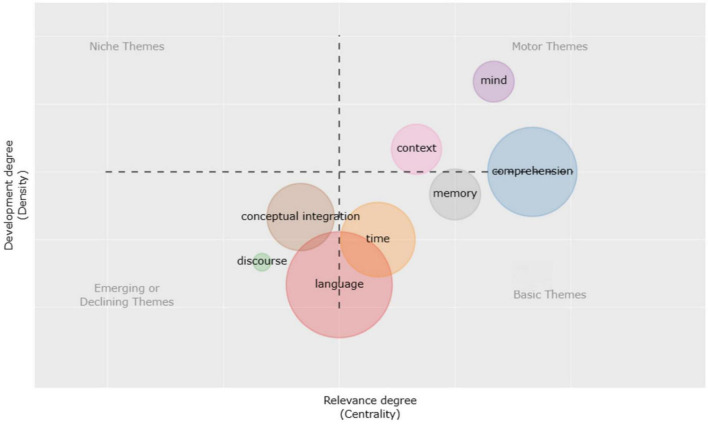
Time slice 1: thematic evolution during 2002–2016.

**FIGURE 8:**
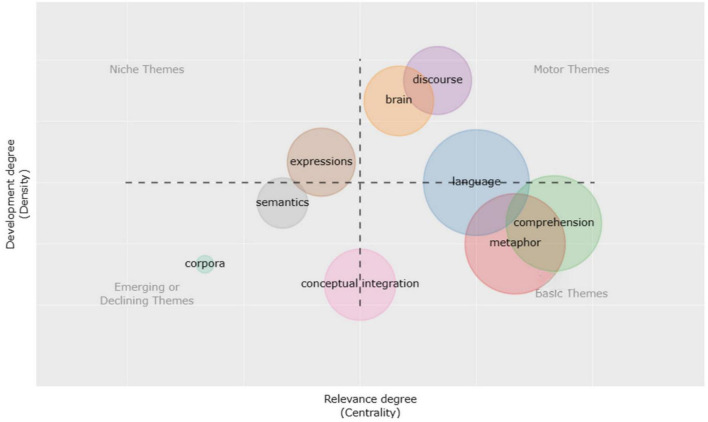
Time slice 2: thematic evolution during 2017–2022.

Figures 7, 8 graphically depict the development of the CM study subjects. 2016 was the dividing line and the two time periods were 2002–2016 and 2017–2022. The topics from 2002 to 2016 may be summed up as “context,” “conceptual integration,” “language,” and “time,” with researchers focusing on conceptual integration. According to the CM hypothesis, metaphor incorporates two cognitive domains, while abstract blending theory theoretically converts two cognitive parts into four mental spaces (Fauconnier and Turner, 1998). It may more precisely characterize people’s psychological processes while using metaphor. In Conceptual Blending Theory, the creation and functioning of conceptual blending are creative. This theory may thus explain not just established mental metaphors but also novel metaphors. Individuals’ daily communication and understanding process is an innovative online mapping and integration process. The relationship between online mapping and fixed mapping is tight. The idea of conceptual blending is comprised of four cognitive domains. According to the theory, the human thinking mode is not a direct, unidirectional, and absolute mapping of the source domain to the target domain but rather a dynamic integration process in which the shared mental schema is a generic space. The two input spaces of the source domain and target domain are bidirectionally mapped to the blending space. Mental space, not the cognitive part, is the fundamental unit of cognitive structure in conceptual blending. Mental space is an abstract area created when individuals think, act, and communicate, intending to achieve local comprehension and action. It is only a transient framework comprised of conceptual aspects like time, belief, desire, possibility, virtuality, place, and reality and depends on the cognitive field, a broader and more fixed knowledge structure associated with a particular cognitive area. It reflects the specific mental schema generated by the cognitive domain and it is dynamic, adaptable, and active throughout the thought process.

From 2017 to 2022, the nature of the mental processes involved in metaphor comprehension was the focus of debate (Stamenković et al., 2019), with dispute focusing on the relative function of common analogical reasoning versus language-specific conceptual blending. The accompanying research indicated that the blending theory framework had explanatory power and practical use.

**FIGURE 9:**
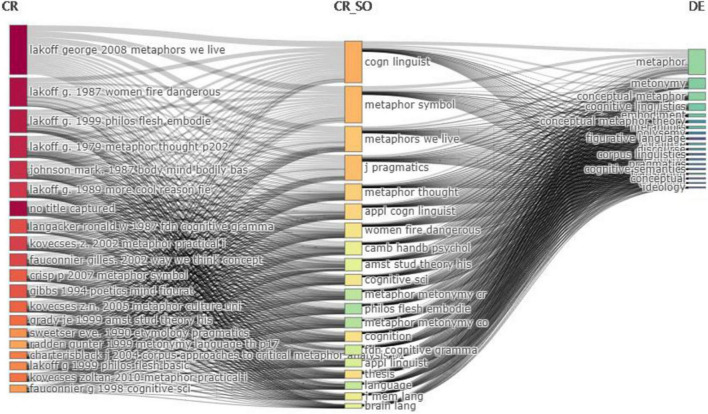
Three-field plot. The middle field is cited sources, the left refers to references, and the right refers to the author’s keywords.

Figure 9 shows that the three fields plot can comprehensively analyze the relationship between measurement indicators of different literature and build a comprehensive network map. According to the statistics, among the periodicals published from 2012 to 2022, *Metaphors We Live By* (Lakoff and Johnson, 2008) was cited first, followed by *Women, Fire and Dangerous Things*: *What Categories Reveal about the Mind* (Lakoff, 1987*).* The middle part of the Three-fields Plot is the Citation Source. *Cognitive Linguistics* ranks first in this field, followed by *Metaphor and Symbol* and *Metaphors We Live By*. *Cognitive Linguistics* is the first citation source, and its corresponding citations are mainly George Lakoff’s books, which shows the authority of George Lakoff, the founder of CM theory, in this field. On the right is the authors’ keyword part. We can see that “metaphor” ranks first on the pyramid, and “metonymy” ranks second. which is consistent with the following Co-occurrence Network (see Figure 11).

**FIGURE 10:**
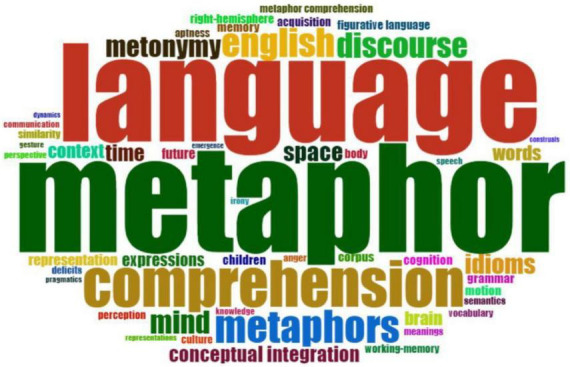
Word cloud. Different words were colored differently, and the size and placement of the phrase denoted their frequency. The size of the colored words depicts the frequency of their occurrence.

**FIGURE 11:**
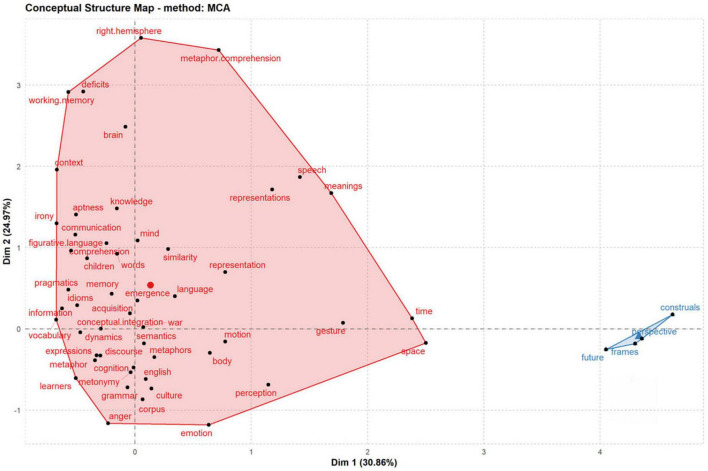
Conceptual structure map. The map is split in half. Clusters are indicated by color, proximity between keywords mean their relationship, the vertex is an illustration of the term in question, and the node’s size is proportional to the frequency with which it appears.

### Research topics in CM

#### Research in the recent two decades

Content analysis was employed to illustrate the CM research issues. Word Cloud, a thematic map of word growth, a conceptual structure map, and the co-occurrence of the author’s keywords were utilized to show study subjects in CM research during the recent two decades.

#### Word cloud

Word clouds are a valuable tool for providing overviews of texts and visualizing relevant words (Herold et al., 2019). Word Cloud was based on the author’s keywords for CM research between 2002 and 2022. With a visual depiction of the Biblioshiny, words with greater volume and keyword density were shown in a bigger and clearer typeface. Word Cloud was used to evaluate commonly used terms in CM research to reveal study subjects. To be more precise, the frequency of usage of a term increased according to its centrality. Based on the author’s keywords, we selected the top 20. First, Figure 10 shows that “metaphor” was the most frequently used term in the authors’ publications, with 143 times in the extracted database, followed by “language (129),” and “comprehension (61).” The number indicates that CM was a vital study issue in cognitive science over the last two decades. The other terms “discourse,” “mind,” and “metonymy” were also used extensively as keywords by writers. It demonstrates that these were essential subjects in the CM field.

#### Conceptual structure map

Researchers may utilize Biblioshiny for Bibliometrix’s Conceptual Structure Map for multiple correspondence analysis (MCA), which aids in sketching a conceptual structure of the area and locating groupings of texts that express similar concepts. Using MCA, one may do a mathematical and graphical analysis of seemingly multivariate data (Greenacre and Blasius, 2006). Figure 11 displays the results of MCA’s clustering on the keywords. The terms “metaphor,” “conception,” and “conceptual integration” often occur in the red set. “Frames,” “future,” and “construal” were added to the blue grouping. Figure 11 shows progress has been made toward developing a significant study subject of CM, and specific research issues around CM have been advanced a fair amount. Metaphors in “time,” “space,” “context,” “emotion,” “anger,” and “expressions” have been analyzed. Multiple fields, such as “pragmatics,” “semantics,” and “memory,” have been thoroughly researched in terms of CM. In addition, the CM research was related to “cognition,” among other things, and not only “discourse,” “metonymy,” “corpus,” and “mind.” Many scholars have conducted studies on metaphor from the perspective of psycholinguistics. Cuccio and Steen (2019) emphasize that attention is a crucial notion in defining deliberateness in metaphor processing because it is the attention we pay to the source domain of a metaphor in working memory that makes a metaphor a deliberately processed metaphor. Gibbs (2013) describes a few complications in psycholinguistic investigations of metaphor and explains the variability of study results. It is common knowledge that engaging in insightful metaphor analysis can be helpful in better comprehending how psychological trauma is conceived. As [Cuccio et al. (2022), p. 1] go, “we need to explain how we use symbols and how we make meanings out of them.” Increasingly, scholars talk about the construal of CM, such as the role of context in the interpretation of CM (Zhao, 2008; Zhao et al., 2020).

#### Co-occurrence of keywords plus

**FIGURE 12:**
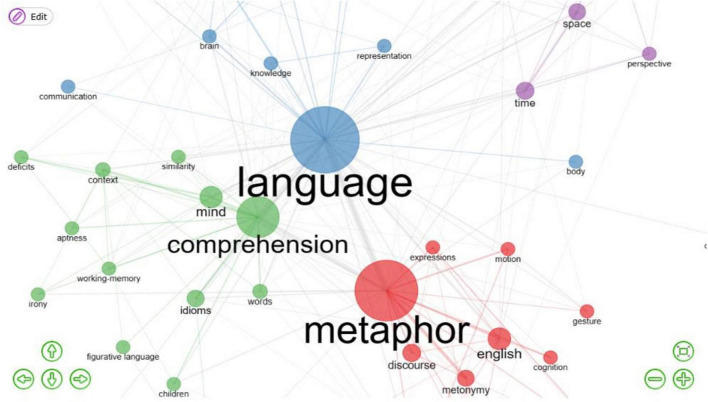
Co-occurrence of keywords plus. The four distinct clusters are blue, green, red, and purple.

Figure 12 presents four distinct clusters: blue, green, red, and purple. The blue cluster focuses on “language,” “representation,” and “communication;” the green cluster emphasizes “comprehension,” “mind,” “idioms,” and “words,” and the red cluster emphasizes “metaphor” and “discourse.” Clustering in purple mostly depends on “time,” “space,” and “perspective.” Consequently, Conceptual Metaphor research emphasizes linguistic theory study, corpus empirical research, and discourse analysis. Critical Metaphor Analysis, also known as CMA, is a method that is typically applied to the process of analyzing metaphors in various critical discourses to reveal the feelings, attitudes, and thoughts that lie behind metaphors. Charteris-Black (2004) proposed “Critical Metaphor Analysis,” which combined pragmatics, cognitive linguistics, and critical discourse analysis. He argued that while cognitive semantics provided a suitable description of how humans comprehended metaphors, the social effect of ideology, culture, and history might give a more persuasive explanation for why specific metaphors were selected in contexts. “Discursive-pragmatic factors, as well as sociolinguistic variation, have to be taken into account to make cognitive analyses more empirically and socially relevant” (Musolff, 2012, p. 301). When it comes to addressing persuasion in text, CM, as it relates to emotion, is a crucial tool because it helps identify the ideological root and persuasive strategy of a given discourse (Ferrari, 2007).

Co-occurrence network development relies heavily on correlation inference. The co-occurrence network has several study and application disciplines, and each color refers to a field. Language is a cognitive tool and a product of human intellect. With the rise of multidisciplinary study, cognitive explanations for grammar creation, semantics, discourse, and metaphor have become widespread, founded on empiricism and cognitive science research. It attempts to explain that language phenomena conform to the human understanding of the brain and thinking, i.e., human language is the product of the human brain, and its construction principle is identical to that of other cognitive domains. Therefore, Figure 12 shows the most significant community, the blue “language.” The closer to the central district, the closer to the “Conceptual Metaphor.” The figure presents that the closest to “language” takes “metaphor” and “comprehension” as the keywords.

The co-occurrence of keywords analysis is a valuable method for constructing a comprehensive framework for comprehending the significant areas of CM study during the past two decades. Figure 12 illustrates a network of co-occurrence between keywords in different types of publications that were established. When two or more of an author’s keywords appeared together, it might indicate how often those terms appeared together in the same publication. Each period was represented as a node, and the greater the node’s size, the more times that the keyword was cited. The greater the thickness of the line that connected two nodes, the more often those terms appeared together.

In the same way, Figure 12 also displays five distinct groups, each representing a different hue. In particular, the terms “discourse” and “metaphor” often co-occurred and were distributed heavily in the red cluster. This suggests that “discourse,” “expressions,” and “English” were prioritized in the CM study, and CM research was practically inseparable from “mind” research. In the center of the purple circle stood the word “time,” but it was disconnected from the surrounding words. However, the connections to “space” and “perspective” were weak. In addition, this analysis discovered that “comprehension” and “metaphor” were often investigated together and that “words” and “idioms” research were linked based on the frequency with which these terms occurred in green nodes. The small size of the nodes and the scarcity of connecting lines suggested that these concerns had not been well-explored.

In conclusion, several terms were used in the investigation of CM. The terms “metaphor,” “language,” “comprehension,” and “English” featured prominently and were among the most often co-occurring in the text. This demonstrated that these issues were central to the CM study. Words like “brain,” “discourse,” and “deficits” also occurred together at the network’s edges, which demonstrated that a wider variety of issues were investigated in CM studies. Despite these variations, it is safe to say that “metaphor,” “language,” and “comprehension” were essential and fundamental study issues, while “knowledge,” “mind,” and “discourse” had an impact on the development of CM and were also widely studied.

### CM research trend

The bibliometric tool of Thematic Evolution and Thematic Trends is employed to predict the directions of potential future CM studies.

#### Thematic evolution

**FIGURE 13:**
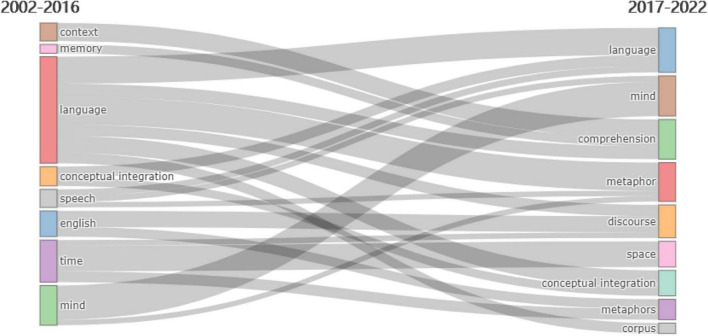
Thematic evolution. Each hue represents a distinct subject, and the size of the rectangle means the depth of study.

Examining Thematic Evolution and Trend Topics may reveal interesting research subjects and possible future orientations. Figure 13 demonstrates the dynamic nature of the metaphor study and the several research topics included. As time went by, “time,” “language,” and “mind” were maintained to be prominent academic areas. Metaphors may offer a practical and memorable method of structuring newly learned terminology. A lexical set is a concept that is well-known to most instructors. A linguistic set groups vocabulary according to a theme, such as “food” or “transportation.” By combining the words and sentences with a metaphorical meaning rather than a literal one, teachers may expand this concept to form “metaphorical sets.” Many scholars have shown their interest in this area. For example, Thomas and McRobbie (2001) emphasize how metaphors may help teachers and students establish a common language of learning. Andreou and Galantomos (2008) investigate the possibility of developing a conceptual curriculum for the instruction of metaphors and idioms in a foreign language setting.

However, throughout 2017–2022, “language” became the primary area of research interest, shifting attention away from “context” and other growing topics like “mind,” “comprehension,” “metaphor,” “discourse,” and “corpus” of CM. Some scholars conducted research into combining critical discourse analysis with self-constructed corpora of diverse genres to determine the underlying ideology metaphor (Semino, 2008; Silvestre-López, 2020). Some academics concentrated on political speech and its associated discourse tactics. For instance, Pilyarchuk and Onysko (2018) found that Trump relied nearly entirely on conventional conceptual metaphors in his talks. Musolff (2011) studied the literary design of the dialog system in Shakespeare’s play and emphasized the general feature of metaphor’s dialogic role, which was further explored concerning the current use of body-based metaphor in political discourse. Koller (2004) explored metaphor and gender in electronic text corpora in the context of the commercial conversation. As for economic discourse, Chen (2018) utilized Wmatrix as a retrieval tool in conjunction with the “MIPVU” to identify and summarize the most prominent conceptual metaphors in economic speech and investigated the significance of metaphors.

Regarding discourse analysis, several efforts emphasized CM used in literary discourse (Zhao and Zhou, 2019). Using the corpus tool Antconc3.2.4w, Zhao et al. (2020) conducted a study on Pearl S. Buck’s novel *Dragon Seed* and pointed out CMT and CBT were concerned with interpreting higher-order concepts such as meaning, language, sign, and representation and their interrelations. They complemented each other and contributed to discourse analysis. CM in literary works might be related to the writer’s cognitive and social contexts. Pearl Buck’s metaphorical thinking was closely related to her experiences in China. It may be extrapolated that these themes have a significant potential for CM research to continue to flourish.

#### Trend topics

**FIGURE 14:**
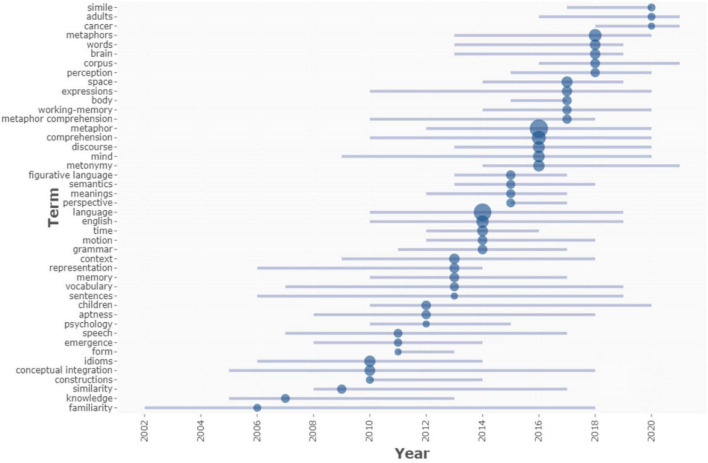
Trend topics by keywords plus. The size of the nodes in Trend Topics represents the total number of publications for a particular topic and the peak popularity of that topic over time.

Figure 14 indicates that, from 2002 to 2013, research subjects were relatively few, but their diversity increased after 2013. The wider the circle in the image, the greater the topic’s popularity among researchers was. Figure 14 shows that 2016 was a banner year for research on “metaphor,” “comprehension,” “discourse,” “mind,” and “metonymy,” as evidenced by the magnitude of the blue node. Between 2002 and 2022, “metaphor” was the most popular subject, appearing 143 times, followed by “language” (129), “comprehension” (61), “synthesis” (61), “metaphors” (43), “English” (38), “discovery” (33), “mind” (33), “meteorology” (28), “idioms” (26), and “space” (25). In 2016, research subjects were the most prevalent and featured the most often. They have been shown, once again, to be central and essential to CM research in recent years, and they may get even more emphasis in the years to come. It happened simultaneously as the Thematic Evolution, which ran from 2017 to 2022. In addition, critical new areas of study, including “metaphor,” “comprehension,” “discourse,” “corpus,” “brain,” “language,” and “mind,” maintained their popularity. The “corpus” of CM studies peaked in 2018 and predictions for its continual fruitfulness in the future were promising. Based on broad corpora, the first kind of investigation establishes the systematicity of conceptual metaphors or summarizes grammatical aspects that conventional metaphor studies cannot notice, compensating for CMT’s deficiencies (Skorczynska and Deignan, 2006). Using CMT as an example, Charteris-Black (2004) proposed a novel research technique that integrated corpus linguistics, critical discourse analysis, and metaphor study to initiate a corpus-based metaphor study and develop new tools for identifying metaphors. Therefore, it is safe to say that “simile,” “adults,” “cancer,” “metaphors,” “words,” “brain,” “corpus,” and “perception” all have promising futures as research areas of CM.

## Conclusion and implication

This study employed a bibliometric technique to investigate 1,257 papers on CM research over the past two decades. The following are significant findings with productivity, content, and citation analysis. First, CM is a cognitive concept and has a widespread academic interest. “Metaphors,” “place,” “discourse,” and “corpus” were the central issues among the various study subjects. “Conceptual integration,” “comprehension,” “language,” and “mind” are also active and popular study topics in CM research. Second, in the past two decades, CM has been a research focus that has included many aspects, including authors, institutions, countries, and sources. Most of the cooperation survey was done with writers and institutions from many nations. The top five countries are Spain, the United States of America, China, Great Britain, and Russia. A rise in the number of academics studying CM suggests that CM research in cognitive linguistics applies to several facets of human cognition. Great Britain and China contributed the most to the growth of CM research, and substantial cooperation and networks were developed among them. These components of CM research are intertwined since the most cited individual contributes to establishing nations, institutions, and papers that significantly impact CM research.

Third, based on an examination of the Thematic Evolution and Trend Topic, we can infer the essential themes in CM research, such as “metaphors,” “discourse,” “space,” and “corpus,” may get greater attention in the future, which aligns with the Thematic Evolution between 2017 and 2022. In addition, “simile,” “adults,” “cancer,” “metaphors,” “words,” “brain,” “corpus,” “perception,” “conceptual integration,” “mind,” and “comprehension” will remain popular themes. The “interdisciplinarity” of CM demonstrates the effect of cognitive context, social context, and other cultural aspects on the framework of CM. The growing number of papers using bibliometric analysis across all disciplines suggests that it meets the desire of researchers who want proper research based on a wealth of literature.

This study will be helpful for beginners in the CM field, allowing them to classify information and find research results of CM quickly so that they may start their research projects. In addition, it may serve as a reference for seasoned researchers to comprehend the progress of CM research over the last two decades, find a suitable collaborator for their present research, and identify research gaps that they may block up in the future.

## Limitation

This study emphasizes the presentation of images and statistics because it is a quantitative study using a bibliometric tool based on data gathered from a database. However, it needs to go more in-depth to complete an evaluation of any specific theme of CM. We urge future research to broaden the study to use a range of more data gathering to examine concerns in CM to create a more thorough comprehension of CM.

## Data availability statement

The original contributions presented in this study are included in this article/supplementary material, further inquiries can be directed to the corresponding author.

## Author contributions

XZ initiated the research idea, instructed YZ to analyze the data using bibliometric software, and co-wrote the article. Under the direction of XZ, YZ gathered and extracted the data and co-wrote the article’s analysis. XCZ contributed to the manuscript’s design, drafting the first part, introduction. All authors participated in revising and approving the version that was submitted.

## Footnotes

1
https://www.bibliometrix.org/home

2
https://clarivate.com/webofsciencegroup/
